# The entorhinal cortex atrophy score for diagnostic distinction between phases of the Alzheimer's disease continuum

**DOI:** 10.1055/s-0046-1829131

**Published:** 2026-10-23

**Authors:** Maria Luiza Vasconcelos Montenegro, Cesar Francisco Penninck de Aguiar Kato, Andressa Rebecah Eloi Batista, Amanda Sampaio de Sá, Francisco Sabino de Oliveira Neto, Maria de Fatima Viana Vasco Aragão, Luziany Carvalho Araujo, Breno José Alencar Pires Barbosa

**Affiliations:** 1 Universidade de Pernambuco, Faculdade de Ciências MédicasRecife PEBrazil; 2 Universidade Federal de Pernambuco, Centro de Ciências MédicasRecife PEBrazil; 3 Universidade Federal de Pernambuco, Hospital das Clínicas, Serviço de RadiologiaRecife PEBrazil; 4 Universidade Federal de Pernambuco, Programa de Pós-Graduação em Neuropsiquiatria e Ciências do ComportamentoRecife PEBrazil; 5 Universidade Federal de Pernambuco, Hospital das Clínicas, Serviço de NeurologiaRecife PEBrazil

**Keywords:** Entorhinal Cortex, Alzheimer Disease, Cognitive Dysfunction, Magnetic Resonance Imaging, Early Diagnosis

## Abstract

**Background:**

Alzheimer's disease (AD) is the most common type of dementia, causing functional impairment in millions of individuals and accounting for a large amount of healthcare costs. The entorhinal cortex appears to be one of the first brain structures affected by AD in its early stages. The Entorhinal Cortex Atrophy (ERICA) score is a visual atrophy score used by neuroradiologists in clinical practice for the diagnosis of AD.

**Objective:**

To analyze the effectiveness of the ERICA score for distinguishing between patients with subjective cognitive decline (SCD), mild cognitive impairment (MCI), and AD dementia.

**Methods:**

A systematic review of the literature was conducted on the Science Direct, PubMed, Virtual Health Library, and Cochrane databases, following the Preferred Reporting Items for Systematic Reviews and Meta-Analyses guidelines. Of 1,413 studies identified, 6 were ultimately included in the present review, comprising 844 participants: 2 retrospective cohort studies and 4 cross-sectional studies. The methodological quality of the studies was assessed using the Newcastle-Ottawa scale. The focus was on the diagnostic performance of the ERICA score in the continuum phases of AD.

**Results:**

The ERICA score had lower sensitivity than the medial temporal lobe atrophy (MTA) score but higher specificity, reinforcing its importance for diagnostic accuracy, especially in contexts of clinical uncertainty. The ERICA score showed reproducibility between examiners similar to or greater than that of the MTA score. There are a few heterogeneous studies in the current literature on the applicability of the ERICA score in clinical practice.

**Conclusion:**

Diagnostic accuracy of the ERICA score is satisfactory in differentiating SCD or MCI from AD dementia.

## INTRODUCTION

Mild cognitive impairment (MCI) is a period of transition between the cognition observed in normal aging and dementia, during the initial phases of Alzheimer's disease (AD). 1 Before MCI, the AD continuum includes the subjective cognitive decline (SCD), in which the patient reports subjective symptoms of cognitive decline, but is independent and performs adequately on cognitive tests. 2 The first signs of neurodegeneration visible on magnetic resonance imaging (MRI) in patients with AD are atrophy of the entorhinal cortex, transentorhinal area, and hippocampus. Hippocampal atrophy has been described as occurring later in the course of the disease than the entorhinal cortex and transentorhinal region. 3 Structured scoring scales are used in clinical settings to standardize the assessment of atrophy across brain structures. 4

One visual scale is the medial temporal lobe atrophy (MTA) score, which assesses the atrophy in the brain region containing the hippocampus, an anatomical structure closely associated with AD. 5 The limitations of the MTA score include being nonspecific to AD, lack of consensus on which cut-off points are considered pathological, and inconsistent protocols for the appropriate coronal cut-off level for image acquisition. 6

Another scale is the Entorhinal Cortex Atrophy (ERICA) score, a quantification of entorhinal cortex atrophy assessed at the level of the mammillary bodies, with scores ranging from 0 (normal) to 3 (severe atrophy). The ERICA score is a good neurodegenerative biomarker for AD in early stages. 3 Another biomarker for cognitive decline is a reduced cerebral blood flow (CBF), which may be significantly related to the spread of AD pathology in the brain. 7 Individual stratification on the AD continuum is essential for adopting personalized therapies at each stage of the disease, with the aim of improving the patient's prognosis. 8

The current systematic review aimed to assess the effectiveness of the ERICA score in evaluating entorhinal cortex atrophy for distinguishing patients with SCD, MCI, and AD dementia. The review also seeks to highlight gaps in the current scientific literature, which can serve as a basis for conducting more detailed studies on this topic.

## METHODS

We conducted a systematic review following the Preferred Reporting Items for Systematic Reviews and Meta-Analyses guidelines. 9 The protocol was previously registered in PROSPERO (CRD42024586741).

### Search strategy

Searches were conducted in the Science Direct, PubMed, Virtual Health Library (VHL), and Cochrane databases for articles published between January 2018 and July 2024. The Boolean operators “AND” and “OR” were used to join search terms, resulting in the following strategies:

- VHL (Health Sciences Descriptors): ( *Comprometimento Cognitivo* OR *Doença de Alzheimer* ) AND ( *RNM* OR *Ressonância Magnética Nuclear* ) AND *Córtex Entorrinal* AND *Atrofia* .
- Science Direct/PubMed/Cochrane (MeSH terms): ( *Cognitive Impairment* OR *Alzheimer Disease* ) AND ( *MRI* OR *Magnetic Resonance Imaging* ) AND *Entorhinal Cortex* AND *Atrophy* .

The terms *ERICA score* and *MTA score* were not included in the search strategies because they were not available in Health Sciences Descriptors and MeSH terms.

### Study selection

The studies were exported to the Rayyan platform (Rayyan Systems Inc.), where duplicate articles were excluded. Two evaluators (MLVM and CFPAK) independently read the titles and abstracts to assess compliance with the established inclusion criteria. A third evaluator (AREB) resolved disagreements found at this stage. The articles were read in full by the three blind evaluators (MLVM, CFPAK, and AREB) to attest to their eligibility ( Figure 1 ).

**Figure 1:**
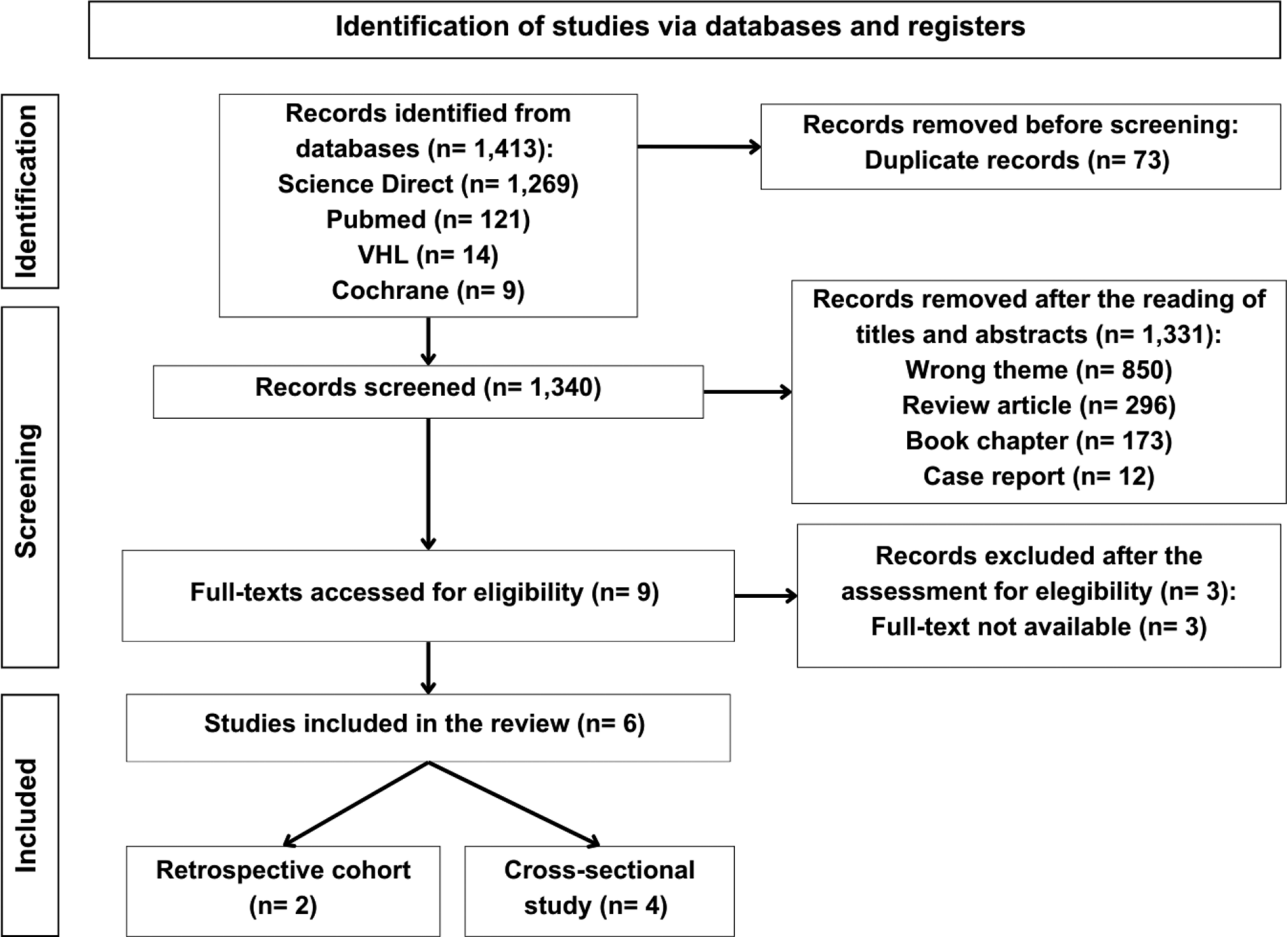
Abbreviation: VHL, Virtual Health Library. Preferred Reporting Items for Systematic Reviews and Meta-Analyses (PRISMA) flowchart for selecting studies included in the systematic review

### Inclusion and exclusion criteria

The inclusion criteria were original studies, such as clinical trials, longitudinal studies, and cross-sectional studies, that evaluated entorhinal cortex atrophy on MRI using the ERICA score ( Figure 2 ) in patients with SCD, MCI, or AD dementia. Only articles published since 2018, when the ERICA score was created, were included. Studies comparing the ERICA score with the MTA score were of particular interest for this review; however, because CBF measured through MRI with pseudocontinuous arterial spin-labeling (ASL) sequence was the second most frequently reported tool, it was also considered. No restrictions were established regarding the research's language or country of origin. Studies that were duplicates, texts that were not open access or could not be obtained through an institutional agreement with the journals, review articles, book chapters, case reports, and dissertations were excluded.

**Figure 2:**
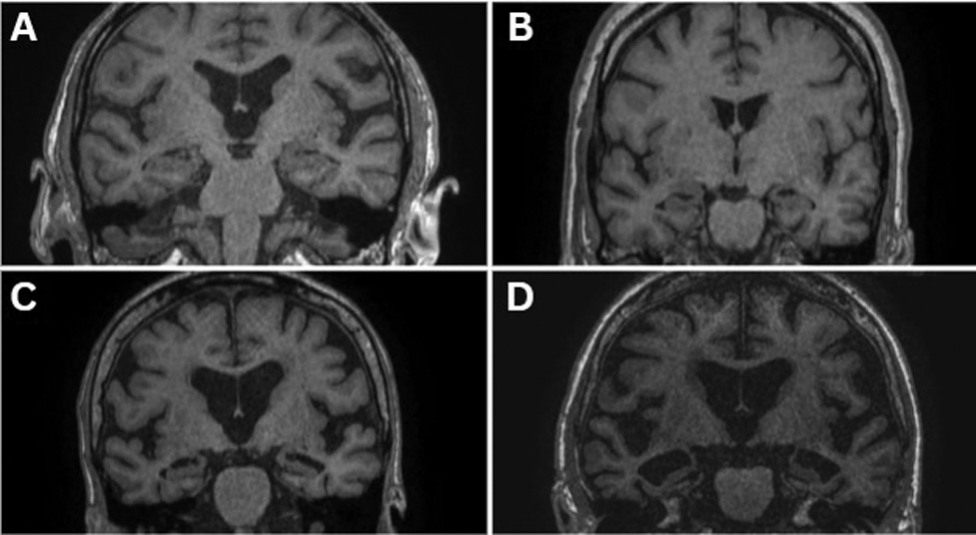
Abbreviation: ERICA, entorhinal cortical atrophy. Source: Images were acquired by the authors in accordance with institutional guidelines and the written informed consent was obtained from all four individuals. Images from magnetic resonance (1,.5T) acquired in T1-weighted sequence, in coronal cut after reconstruction. ( **A** ) Normal volume of the entorhinal cortex and the parahippocampal gyrus (ERICA score 0). ( **B** ) Mild atrophy of the entorhinal cortex and the parahippocampal gyrus and widening of the collateral groove (ERICA score of 1). ( **C** ) Moderate atrophy of the entorhinal cortex, moving away from the tentorium cerebelli (ERICA score of 2). ( **D** ) Severe atrophy of the entorhinal cortex and parahippocampal gyrus, with wide separation to the tentorium cerebelli (ERICA score of 3).

### Data extraction and assessment of methodological quality

Data from the articles were transposed into a Google Sheets spreadsheet. The methodological quality of the studies was assessed using the Newcastle-Ottawa scale. 10 The items measured by this scale relate to participant selection, the degree of comparability between groups, and the assessment of the outcome or exposure in cohort and case-control studies, respectively. The methodological quality of the studies was judged to be satisfactory, achieving at least six stars. 10 No article was excluded based on this criterion. The classification according to the Newcastle-Ottawa scale was also presented in accordance with the quality definitions of the Health Research and Quality Agency, which subdivide the standards into good, fair, and poor. 11 Two evaluators (MLVM and CFPAK) conducted this review stage independently, and any inconsistencies were shared to reach a common decision for all papers.

## RESULTS

### Summary of searches

Initially, 1,413 articles were identified. After removing duplicates, 1,340 studies remained; 1,331 were excluded because they did not meet the inclusion criteria. Nine articles were chosen for the full reading stage to verify the eligibility, and six were ultimately included in the present review: two retrospective cohort studies and four cross-sectional studies ( Figure 1 ).

In the study by Traschütz et al., 12 the median follow-up time was 28 months, with the median time to conversion from MCI to AD dementia being 24 months. In the study by Roberge et al., 13 the total follow-up time for patients was up to 3 years, and the time of conversion from the MCI phase to AD dementia was not reported.

### Study characteristics

The studies used MRI at a field strength of 1.5 to 3.0 tesla to acquire T1-weighted images with slice thicknesses of 1.0 to 1.2 mm. Regarding the diagnosis of probable AD, some studies 3 12 13 used biomarkers, among other criteria, while others 14 15 16 used clinical and neuroimaging ( Table 1 ). The sample sizes of the studies included ranged from 60 to 328 participants, for a total of 844 patients. The composition of the control group varied between individuals with SCD 3 14 and people considered cognitively healthy 15 16 ( Table 2 ).

**Table 1:** Main characteristics of the studies included in the review

Main author (year of publication)	Country	Study design	MRI (Tesla)	Type of cuts	Thickness	Sequence	Biomarker-based diagnosis
Enkirch et al. (2018) 3	Germany	Cross-sectional	3T	Coronal cuts aligned with the mammillary bodies	1 mm	3D T1-weighted	Yes
Thomas et al. (2019) 15	India	Cross-sectional	3T	Coronal sections at the level of the mammillary bodies	1 mm	3D T1-weighted	No
Traschütz et al. (2020) 12	Germany	Retrospective cohort	3T	Coronal sections aligned with the brain stem	1 mm	3D T1-weighted	Yes
Roberge et al. (2021) 13	Canada	Retrospective cohort	1.5T or 3T	Coronal sections aligned with the brain stem	1 mm	3D T1-weighted	Yes
Ikanga et al. (2023) 16	Democratic Republic of the Congo	Cross-sectional	1.5T	Oblique coronal sections orthogonal to the main axis of the hippocampus	1.2 mm	3D T1-weighted	No
Essien et al. (2023) 14	United States	Cross-sectional	1.5T or 3T	Coronal sections at the level of the mammillary bodies	1 mm	3D T1-weighted	No

Abbreviations: MRI, magnetic resonance imaging; T, tesla; 3D, three-dimensional.

**Table 2:** Demographic characteristics of the population of all six studies

Main author (year of publication)	Sample size	Groups (N)	Women (%)	Mean age in years
Enkirch et al. (2018) 3	120	SCD (60) AD (60)	SCD (47%) AD (33%)	SCD (69.4) AD (72.4)
Thomas et al. (2019) 15	60	HC (21) MCI (20) AD (19)	HC (47.6%) MCI (45%) AD (42.1%)	HC (64.5) MCI (66.7) AD (66.6)
Traschütz et al. (2020) 12	200	SCD (60) MCI (80) AD (60)	SCD (47%) MCI (38%) AD (33%)	SCD (69.4) MCI (72.9) AD (72.4)
Roberge et al. (2021) 13	61	aMCI (61)	aMCI (44.3%)	aMCI (68.6)
Ikanga et al. (2023) 16	75	HC (29) Probable AD (46)	HC (62%) Probable AD (45.6%)	HC (71.2) Probable AD (73.3)
Essien et al. (2023) 14	328	SCD (38) MCI (172) AD (118)	SCD (42.1%) MCI (58.1%) AD (60.2%)	SCD (67) MCI (74) AD (78)

Abbreviations: AD, Alzheimer's disease; aMCI, amnestic mild cognitive impairment; HC, healthy controls; MCI, mild cognitive impairment; SCD, subjective cognitive decline.

Enkirch et al. 3 included only patients diagnosed with AD dementia who had either altered biomarkers in cerebrospinal fluid (CSF) or amyloid positron emission tomography (PET). For their cohort of patients with SCD, Enkirch et al. 3 also prioritized patients diagnosed using CSF biomarker analysis: Ab40, Ab42, total tau (t-tau), and phosphorylated tau (p-tau). Traschütz et al. 12 analyzed the CSF biomarkers (Ab42, t-tau, p-tau, Ab42/40 ratio, and Ab42/p-tau) of patients with SCD, MCI, and AD dementia. Only 65% of patients with MCI had available CSF biomarkers, which were positive for amyloid, tau, and p-tau in 70%, 62%, and 68%, respectively; however, these rates were not reported for SCD and AD dementia. 12 For Roberge et al., 13 the presence of CSF biomarkers (Ab1–42, p-tau, and t-tau) was necessary to diagnose AD pathology; however, only 21.3% of patients had positive CSF biomarkers, and the proportions of MCI and AD dementia in this group of patients with positive biomarkers were not distinguished.

Regarding inter-rater reliability, the study by Enkirch et al. 3 showed a consensus for the ERICA score similar to that of the MTA score, with Cohen k = 0.88 (95% confidence interval [CI]: 0.80–0.97) versus 0.91 (95% CI: 0.87–0.94), respectively; in the study by Roberge et al., 13 Cohen k estimates for the ERICA and MTA scores were, respectively, at 0.43 (95% CI: 0.25–0.61) versus 0.27 (95% CI: 0.15–0.40) for the right hemisphere and 0.56 (95% CI: 0.39–0.72) versus 0.36 (95% CI: 0.23–0.50) for the left, showing some superiority of the ERICA score; and Thomas et al. 15 reported a Cohen k = 0.78 for the right hemisphere and 0.77 for the left for the ERICA score. The other studies 12 13 14 15 16 either did not use two examiners or did not perform this type of comparative analysis.

### Diagnostic performance

Table 3 describes the diagnostic performance of the ERICA score across the studies included.

**Table 3:** Discriminative performance of ERICA and comparisons with other tools across the clinical stages of Alzheimer's disease

Study (year)	Clinical comparison	Imaging marker (cutoff)	AUC	95% CI	*p* -value	*p* -value* (AUC COMPARASION)	Sensitivity (95% CI)	Specificity (95% CI)	Clinical highlights
Enkirch et al. (2018) 3	SCD versus AD	MTA score (N/A)	0.82	0.74–0.89	N/A	*p* < 0.001	57% (43–69)	92% (82–97)	ERICA score: superior sensitivity, specificity and AUC.
		ERICA score (≥ 2)	0.93	0.88–0.98	N/A		**83% (71–92)**	**98% (91–100)**	
Essien et al. (2023) 14	SCD versus AD	MTA score (N/A)	0.80	0.71–0.89	N/A	*p* = 0.28	**84% (77–91)**	66% (51–81)	ERICA score: superior specificity for rule- in diagnosis.
		ERICA score (2)	0.83	0.76–0.90	N/A		69% (61–78)	**87% (76–98)**	
Traschütz et al. (2020) 12	MCI versus AD	MTA score (N/A)	0.62	0.53–0.71	N/A	*p* = 0.006	65% (N/A)	46% (N/A)	ERICA score: higher sensitivity, specificity and AUC in the prodromal phase.
		ERICA score (N/A)	0.71	0.61–0.89	N/A		**83% (N/A)**	**59% (N/A)**	
Essien et al. (2023) 14	MCI versus AD	MTA score (N/A)	0.73	0.68–0.79	N/A	*p* = 0.25	**84% (77–91)**	54% (47–62)	ERICA score: higher specificity. MTA score: higher sensitivity.
		ERICA score (3)	0.71	0.65–0.77	N/A		69% (61–78)	**66% (59–73)**	
Thomas et al. (2019) 15	MCI versus AD	ERICA score (N/A)	0.83	N/A	*p* < 0.001	N/A	N/A	N/A	ERICA score: stronger predictor than CBF.
		CBF (N/A)	0.58	N/A	*p* = 0.339		N/A	N/A	
Traschütz et al. (2020) 12	SCD versus MCI	MTA score (N/A)	0.72	0.64–0.81	N/A	*p* = 0.52	40% (N/A)	92% (N/A)	ERICA score: higher specificity.
		ERICA score (N/A)	0.74	0.66–0.82	N/A		41% (N/A)	**98% (N/A)**	
Enkirch et al. (2018) 3	SCD versus MCI	MTA score (N/A)	0.65	0.54–0.77	N/A	*p* = 0.801	N/A	N/A	ERICA score: low discriminatory power for early transition, similar to MTA score.
		ERICA score (≥ 2)	0.66	0.54–0.78	N/A		N/A	N/A	
Essien et al. (2023) 14	SCD versus MCI	MTA score (N/A)	0.61	0.51–0.71	N/A	N/A	N/A	N/A	ERICA score: poor discriminatory power between early phases, similar to MTA score.
		ERICA score (N/A)	0.64	0.55–0.73	N/A		N/A	N/A	
Thomas et al. (2019) 15	Control versus AD	ERICA score (N/A)	0.88	N/A	*p* < .001	N/A	N/A	N/A	ERICA score: stronger predictor than CBF.
		CBF (N/A)	0.81	N/A	*p* < .001		N/A	N/A	
Thomas et al. (2019) 15	Control versus MCI	ERICA score (N/A)	0.57	N/A	*p* = 0.493	N/A	N/A	N/A	CBF: stronger predictor than ERICA score.
		CBF (N/A)	**0.77**	N/A	*p* = 0.002		N/A	N/A	

Abbreviations: AD, Alzheimer's disease; AUC, area under the curve; CBF, cerebral blood flow; CI, confidence interval; ERICA, entorhinal cortical atrophy; MCI, mild cognitive impairment; MTA, medial temporal lobe atrophy; N/A, not applicable; SCD, subjective cognitive decline.

Notes: The first *p* -value refers to the statistical significance of the AUC, whereas the second *p* -value* refers to the statistical comparison between the AUCs of different methods.

### Control versus mild cognitive impairment

In the study that compared healthy controls with those with MCI, the ERICA score did not significantly differentiate these two groups of patients, being inferior to CBF with an area under the curve (AUC) of 0.57 ( *p* = 0.493) versus 0.77 ( *p* = 0.002). When combined, these two techniques showed good predictive value (AUC = 0.78; *p* = 0.002) for distinguishing healthy individuals from the MCI group, but the predictive value did not differ from that of CBF alone. 15

### Subjective cognitive decline versus mild cognitive impairment

Enkirch et al. 3 reported an AUC of 0.66 (95% CI: 0.54–0.78) for the ERICA score and 0.65 (95% CI: 0.54–0.77) for the MTA score ( *p* = 0.801). Traschütz et al. 12 found there was no significant difference in the discrimination between SCD and MCI for the ERICA score (AUC = 0.74; 95% CI: 0.66–0.82), MTA score (AUC = 0.72; 95% CI: 0.64–0.81; *p* = 0.52), hippocampal volumetry (AUC = 0.78; 95% CI: 0.71–0.86; *p* = 0.24), or volumetry of the anterior medial temporal lobe (AUC = 0.71; 95% CI: 0.63–0.80; *p* = 0.59). Moreover, the ERICA score had a sensitivity of 41% and a specificity of 98%, compared with the MTA score, which had a sensitivity of 40% and a specificity of 92%. 12 In contrast, Essien et al. 14 found that the ERICA score (AUC = 0.64; 95% CI: 0.55–0.73), MTA score (AUC = 0.61; 95% CI: 0.51–0.71), and normalized total hippocampal volume (NTHV) (AUC = 0.66; 95% CI: 0.55–0.77) were not good discriminators between these two clinical entities.

### Control versus Alzheimer's disease

When evaluating healthy controls compared with AD dementia patients, the diagnostic performance of the ERICA score (AUC = 0.88; *p* < .001) was superior to that obtained by the CBF technique (AUC = 0.81; *p* < .001), but even better when these two tools were used simultaneously (AUC = 0.95; *p* < .001). 15 Another study found that 31% of healthy controls and 93.5% of patients diagnosed with probable AD dementia had abnormalities on the ERICA score, compared with 44.8% and 91.3% with changes on MRI, as measured by the MTA score. 16

### Subjective cognitive decline versus Alzheimer's disease

In studies that evaluated the diagnostic capability to discriminate between SCD and AD, Enkirch et al. 3 revealed that an ERICA score of 2 or greater was more specific (98%; 95% CI: 91–100) and more sensitive (83%; 95% CI: 71–92) than the MTA score, which had specificity of 92% (95% CI: 82–97) and sensitivity of 57% (95% CI: 43–69) to differentiate SCD from AD dementia. The ERICA score of 2 or greater reached an AUC of 0.93 (95% CI: 0.88–0.98), significantly greater ( *p* < 0.001) than the 0.82 (95% CI: 0.74–0.89) achieved by the MTA score. 3

Essein et al. 14 demonstrated that the ERICA score cutoff value of 2 had higher specificity than the MTA score and NTHV, of 87% (95% CI: 76–98) versus 66% (95% CI: 51–81) versus 74% (95% CI: 60–88) respectively. However, this same ERICA score cutoff had lower sensitivity (69%; 95% CI: 61–78) than the MTA score (84%; 95% CI: 77–91) and NTHV (84%; 95% CI: 76–90). It was also shown that the ERICA score (AUC = 0.83; 95% CI: 0.76–0.90), MTA score (AUC = 0.80; 95% CI: 0.71–0.89), and NTHV (AUC = 0.83; 95% CI: 0.74–0.91) performed better than chance with no significant differences comparing ERICA versus MTA ( *p* = 0.28), NTHV versus ERICA ( *p* = 0.90) and NTHV versus MTA ( *p* = 0.37). 14

### Mild cognitive impairment versus Alzheimer's disease

In differentiating MCI and dementia due to AD, Essien et al. 14 found values better than chance for the ERICA score (AUC = 0.71; 95% CI: 0.65–0.77), MTA score (AUC = 0.73; 95% CI: 0.68–0.79), and NTHV (AUC = 0.75; 95% CI: 0.69–0.81), but with no significant difference between the ERICA and MTA ( *p* = 0.25), NTHV and ERICA ( *p* = 0.12) and NTHV and MTA ( *p* = 0.47). It was reported that an ERICA score cutoff value of 3 had sensitivity of 69% (95% CI: 61–78), which was lower than the MTA score (84%, 95% CI: 77–91) and similar to NTHV (71%; 95% CI: 62–79). Nevertheless, the ERICA score cutoff value of 3 had a specificity of 66% (95% CI: 59–73), which was higher than that of the MTA score (54%; 95% CI: 47–62) and similar to that of NTHV (67%; 95% CI: 59–74). 14

In turn, Traschütz et al. 12 showed that the ERICA score (AUC = 0.71; 95% CI: 0.61–0.89) distinguished better subjects with MCI than those with AD dementia compared with the MTA score (AUC = 0.62; 95% CI: 0.53–0.71; *p* = 0.006), however ERICA score discrimination was similar to hippocampus or posterior medial temporal lobe volumetry (both AUC = 0.68; 95% CI: 0.59–0.77; *p* > 0.47). This same project also reported sensitivities and specificities of 83% and 59% for the ERICA score, and 65% and 46% for the MTA score, respectively, to differentiate patients with MCI from those with AD dementia. 12

Thomas et al. 15 reported a higher AUC for the ERICA score (AUC = 0.83; *p* < .001) to distinguish MCI from AD dementia compared with regional mean CBF (AUC = 0.58; *p* = 0.339). When these two techniques were used together, the AUC was reduced (AUC = 0.82; *p* < 0.001). 15

### Prognostic performance

Of the selected studies, two 12 13 presented data on the ERICA score in the conversion of MCI to AD dementia in longitudinal analyses. Traschütz et al. 12 found a sensitivity of 53% and specificity of 86% for the abnormal ERICA score, but a sensitivity of 63% and specificity of 86% for the MTA score to predict conversion from MCI to AD dementia ( Table 4 ). A similar discriminative performance between methods was also reported, with an AUC of 0.70 for the ERICA score versus an AUC of 0.71 for the MTA score ( *p* = 0.91) and compared with an AUC of 0.74 for the anterior medial temporal lobe volumetry ( *p* = 0.67). 12

**Table 4:** Prognostic value of the ERICA score compared with the MTA score

STUDY (YEAR)	CLINICAL COMPARISON	IMAGING BIOMARKER (CUTOFF)	AUC	95% CI	*P* -VALUE	*P* -VALUE* (AUC COMPARISON)	SENSITIVITY (95% CI)	SPECIFICITY (95% CI)	CLINICAL HIGHLIGHT
Traschütz et al. (2020) 12	MCI VS. AD	MTA score (N/A)	0.71	N/A	N/A	*p* = 0.91	**63% (N/A)**	86% (N/A)	ERICA score: Specificity similar to that of MTA. MTA score: Higher Sensitivity.
		ERICA score (≥ 2)	0.70	N/A	N/A		53% (N/A)	86% (N/A)	
Roberge et al. (2021) 13	MCI VS. AD	MTA score (≥ 2 if ≤ 75 yo or ≥ 3 if > 75 yo)	0.64	N/A	*p* = 0.053	N/A	**69% (41–89%)**	60% (44–74%)	ERICA score: Higher Specificity and AUC. MTA score: Higher Sensitivity.
		ERICA score (≥ 2)	**0.67**	N/A	*p* = 0.015		56% (30–80%)	**78% (63–89%)**	

Abbreviations: AD, Alzheimer's disease; AUC, area under the curve; CI, confidence interval; ERICA, entorhinal cortical atrophy; MCI, mild cognitive impairment; MTA, medial temporal lobe atrophy; N/A, not applicable; SCD, subjective cognitive decline; yo, years old.

In the analysis of the conversion of patients with MCI to AD dementia, Roberge et al. 13 showed sensitivity and specificity, respectively, of 56% (95% CI: 30–80%) and 78% (95% CI: 63–89%) ( *p* = 0.02) and an AUC of 0.67 ( *p* = 0.015) for the ERICA score (≥ 2–3). The MTA score (≥ 2 for ≤ 75 years old, or ≥ 3 for > 75 years old), in turn, had sensitivity of 69% (95% CI: 41–89%), specificity of 60% (95% CI: 44–74%), and AUC of 0.64 ( *p* = 0.053). 13

### Methodological quality of studies

According to the Newcastle-Ottawa scale, the 2 cohort studies 12 13 received 7 stars, while the 4 cross-sectional studies 3 14 15 16 received at least 8 stars, as shown in Figure 3 .

**Figure 3:**
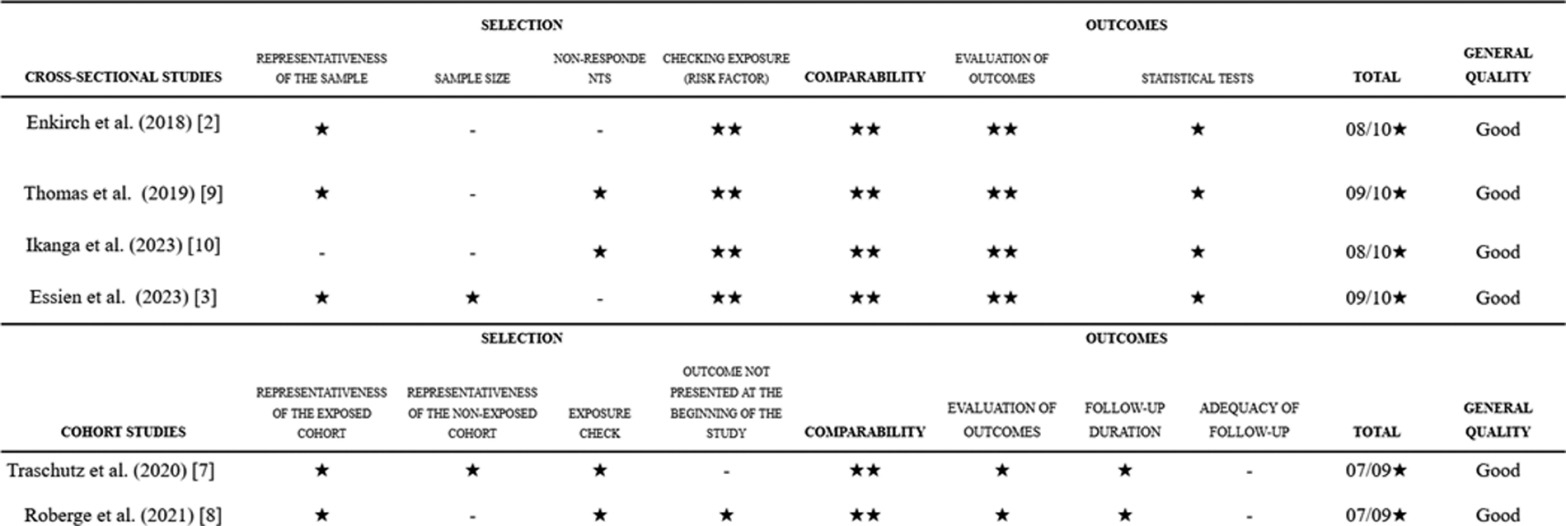
Assessment of the quality of studies according to the Newcastle-Ottawa scale.

## DISCUSSION

The present study aimed to investigate the role of the ERICA score as a diagnostic tool for SCD, MCI, and AD dementia. Overall, the ERICA score demonstrated discriminative performance significantly above chance for distinguishing healthy controls from AD dementia, MCI from AD dementia and SCD from AD dementia. Compared with ERICA score, CBF better differentiated healthy controls from those with MCI. There was no statistically significant difference observed between ERICA score and MTA score in distinguishing SCD and MCI. As an additional finding, two 12 13 of the six articles discussed the ERICA score's predictive power in converting MCI to AD dementia.

The MTA score is one of the most used neuroimaging tools in clinical practice for diagnosing AD. 17 One study found that the MTA score was negatively correlated with the severity of the Mini-Mental State Examination in a statistically significant way. 18 Another research concluded that the MTA score was able to discriminate which patients would develop AD or remain with MCI. 19

A study conducted in Peru found that the MTA score, when combined with the visual scale of posterior atrophy, had greater discriminative power between healthy individuals and those with AD than the MTA score alone. 20 The applicability of the MTA score for the early diagnosis of AD is limited, as it cannot distinguish individuals in the early stages of the AD continuum. 21

In parallel, the ERICA score is designed to assess atrophy of the entorhinal cortex, the brain region most affected early in patients with AD. 22 Regarding inter-rater reliability, two studies 3 15 showed similar rates between the ERICA and MTA scores, and in another study, 13 the ERICA score had a higher rate but remained moderate. The ERICA score outperformed the MTA score in distinguishing SCD from AD dementia and MCI from AD dementia in two studies. 3 12

Alzheimer's disease dementia is co-pathologically associated with cerebrovascular disease in up to 50% of cases, since the deposition of β-amyloid protein in brain tissue induces damage to the vessel walls and, consequently, a reduction in CBF. 23 In addition, CBF is closely associated with neuronal metabolic demand, and reductions in its levels may represent early synaptic dysfunction and cerebral hypometabolism, which are usually present in the early stages of Alzheimer's disease. 24 A longitudinal study in patients with SCD showed that amyloid deposition in AD and cerebrovascular disease are concurrent conditions, since reduced regional CBF contributes to increased amyloid pathology. 25 Other research showed that hypertension may cause cognitive decline through white matter lesions associated with reduced CBF. Moreover, this vascular damage is an independent risk factor for increased tau pathology and entorhinal cortex atrophy. 26 Compared with the ERICA score, CBF distinguished better control subjects from those with MCI, whereas the ERICA score performed better in differentiating control individuals from those with AD dementia and MCI subjects from those with AD dementia. 15

The ERICA score is a useful tool in structural MRI analysis, with performance levels comparable to widely established approaches such as automated volumetry. The use of visual brain atrophy scales, such as ERICA and MTA scores, enables the diagnosis of different stages of the AD continuum in institutions with fewer resources, given their lower complexity and lower cost compared with automated volumetric methods. 3 12 14 In two studies, 12 13 only one found that the ERICA score achieved greater specificity, compared with MTA score, in converting MCI to AD.

The diagnostic performance of the ERICA score is limited by both methodological and biological factors. Its sensitivity is influenced by reader training and by the fact that entorhinal cortex atrophy is not uniformly present in all patients with AD. Disease stage, interindividual vulnerability, and phenotypic variability contribute to the absence of visually appreciable entorhinal involvement in a substantial proportion of cases, particularly in early or non-amnestic presentations. 27 This limitation is shared by all visually-based atrophy scales. The ERICA score cannot reliably capture the full spectrum of AD-related neurodegeneration, and a normal ERICA score should not be interpreted as evidence against the diagnosis.

From a clinical perspective, ERICA is not intended to replace established visual scales such as MTA, global cortical atrophy (GCA), or posterior atrophy (Koedam). It may serve as an adjunct within a comprehensive visual assessment strategy, by providing a reproducible, low-cost, and readily available method for evaluation of the entorhinal cortex. When interpreted alongside other visual scales, ERICA may offer incremental anatomical insight, particularly in diagnostically challenging, without being regarded as a definitive diagnostic marker.

The diagnosis of AD has advanced significantly, notably with the ATN-I criteria, which introduce biomarkers related to inflammatory processes. 28 Structural neuroimaging methods are nonspecific markers of neurodegeneration and add considerable diagnostic value in scenarios of low availability of amyloid and tau biomarkers. Despite the emergence of new serum biomarkers, neuroimaging remains relevant in the investigation of dementia cases. 28

The limitations of the present study lie in the small number of studies dedicated to the ERICA score in MRI images; therefore, it was impossible to conduct a meta-analysis. Not all research studies have used specific biomarkers (i.e., amyloid or p-tau) as diagnostic criteria for AD, which constitute the most reliable way to identify the presence of this disease. 28 The predefined inclusion criteria were retained, considering that research on the ERICA score is still in its early stages in the literature. Including only articles in which AD was diagnosed using biomarkers would limit the number of eligible articles, potentially weakening the clinical application of the present study's findings. However, including patients not diagnosed with biomarkers, especially those considered healthy controls without available amyloid status, may obscure the results of this review, since biomarker diagnosis is essential for inclusion in the AD continuum. 29 Another limiting factor is the methodology of the studies included, which were cross-sectional and retrospective, highlighting the need for new prospective longitudinal research to more accurately analyze the diagnostic performance of the ERICA score.

As the main study's strength, we performed a comprehensive, thorough search for the first time, which included studies with good methodological quality that addressed the use of the ERICA score as a diagnostic tool for the AD continuum phases. This is relevant considering that specific AD biomarkers are not widely available in low and middle-income countries, 30 31 where MRI still plays a significant role in the diagnostic workup of patients with MCI and dementia.

Overall, the ERICA score showed good accuracy in distinguishing between stages such as SCD or MCI and consolidated AD dementia. Compared with MTA and automated hippocampal volumetry, the ERICA score did not demonstrate superiority over these tools. Despite its lower sensitivity, the ERICA score stands out for its higher specificity, making it an important marker in scenarios where greater diagnostic certainty is required. Therefore, we encourage the use of the ERICA score as a complementary tool to MTA score, increasing specificity in the diagnosis of AD dementia, especially in scenarios of clinical uncertainty.
